# An Alternative Approach for Estimating the Accuracy of Colposcopy in Detecting Cervical Precancer

**DOI:** 10.1371/journal.pone.0126573

**Published:** 2015-05-11

**Authors:** Kalatu R. Davies, Scott B. Cantor, Dennis D. Cox, Michele Follen

**Affiliations:** 1 Department of Health Services Research, The University of Texas MD Anderson Cancer Center, Houston, Texas, United States of America; 2 Department of Statistics, Rice University, Houston, Texas, United States of America; 3 Department of Obstetrics & Gynecology, Brookdale University Hospital & Medical Center, Brooklyn, New York, United States of America; University of Utah Health Sciences Center, UNITED STATES

## Abstract

**Introduction:**

Since colposcopy helps to detect cervical cancer in its precancerous stages, as new strategies and technologies are developed for the clinical management of cervical neoplasia, precisely determining the accuracy of colposcopy is important for characterizing its continued role. Our objective was to employ a more precise methodology to estimate of the accuracy of colposcopy to better reflect clinical practice.

**Study design:**

For each patient, we compared the worst histology result among colposcopically positive sites to the worst histology result among all sites biopsied, thereby more accurately determining the number of patients that would have been underdiagnosed by colposcopy than previously estimated.

**Materials and Methods:**

We utilized data from a clinical trial in which 850 diagnostic patients had been enrolled. Seven hundred and ninety-eight of the 850 patients had been examined by colposcopy, and biopsy samples were taken at colposcopically normal and abnormal sites. Our endpoints of interest were the percentages of patients underdiagnosed, and sensitivity and specificity of colposcopy.

**Results:**

With the threshold of low-grade squamous intraepithelial lesions for positive colposcopy and histology diagnoses, the sensitivity of colposcopy decreased from our previous assessment of 87.0% to 74.0%, while specificity remained the same. The drop in sensitivity was the result of histologically positive sites that were diagnosed as negative by colposcopy. Thus, 28.4% of the 798 patients in this diagnostic group would have had their condition underdiagnosed by colposcopy in the clinic.

**Conclusions:**

In utilizing biopsies at multiple sites of the cervix, we present a more precise methodology for determining the accuracy of colposcopy. The true accuracy of colposcopy is lower than previously estimated. Nevertheless, our results reinforce previous conclusions that colposcopy has an important role in the diagnosis of cervical precancer.

## Introduction

Cervical cancer can be prevented if abnormal cells are detected and treated in the precancerous stages [1]. Thus, the current standard of care for detection of cervical neoplasia, a Papanicolaou smear followed by colposcopically directed biopsy [2], requires a high degree of diagnostic accuracy. To improve diagnostic accuracy, physicians often biopsy multiple sites that are judged clinically suspicious by colposcopy [3].

Recent studies have evaluated accuracy in terms of level of agreement and correlation of the overall results of colposcopy to corresponding biopsy with outcomes ranging from a sensitivity of 70% to 98% and specificity of 45% to 90%, showing greater disease thresholds resulting in higher sensitivity and lowered specificity [2–9]. Boicea et al. found high accuracy and correlation between colposcopy and biopsy with a subsequent sensitivity of 83.6% [4]. In a study by Karimi-Zarchi et al. [6], a pathologist blinded to the results of colposcopy read and recorded the results of colposcopy and biopsy, finding a sensitivity of 70.9% and specificity of 77.9% for colposcopy. Benedet et al. found satisfactory agreement between the colposcopic diagnosis and accompanying biopsies with a sensitivity of 90.3% and a specificity of 57.3% [10].

In the parent study [11] involving development of optical technologies for detecting cervical neoplasia, we included provisions for biopsying colposcopically normal sites, thereby creating a rich database of diagnoses by site for each patient. This additional sampling enabled us to identify patients who would have been underdiagnosed in the clinic. This study was unique in the sense that for each patient we had colposcopy and biopsy results from multiple sites in the cervix, including those diagnosed as normal and abnormal by colposcopy, allowing us to get a more precise measure of the accuracy of colposcopy in selecting sites for biopsy for the various participant subgroups. A recent study by Wentzensen et al. showed that the sensitivity of detection of high-grade lesions increases with the number of biopsies [12]. The current study was therefore aimed at developing a methodology for better assessing the accuracy of visual examination by clinicians in selecting sites for biopsy.

In a previous analysis [5], Cantor et al. summarized results in terms of each patient’s worst colposcopy result and worst histology finding. In the current study, by identifying colposcopically negative sites that were found to be positive for cervical neoplasia by histology, we sought to evaluate the diagnostic accuracy of colposcopy in terms of the percentage of patients with cervical precancer that would have been underdiagnosed by colposcopy in clinic. The proposed method is an effort to evaluate colposcopy using a paradigm that is more reflective of actual clinical practice.

## Methods

**Fig 1:**
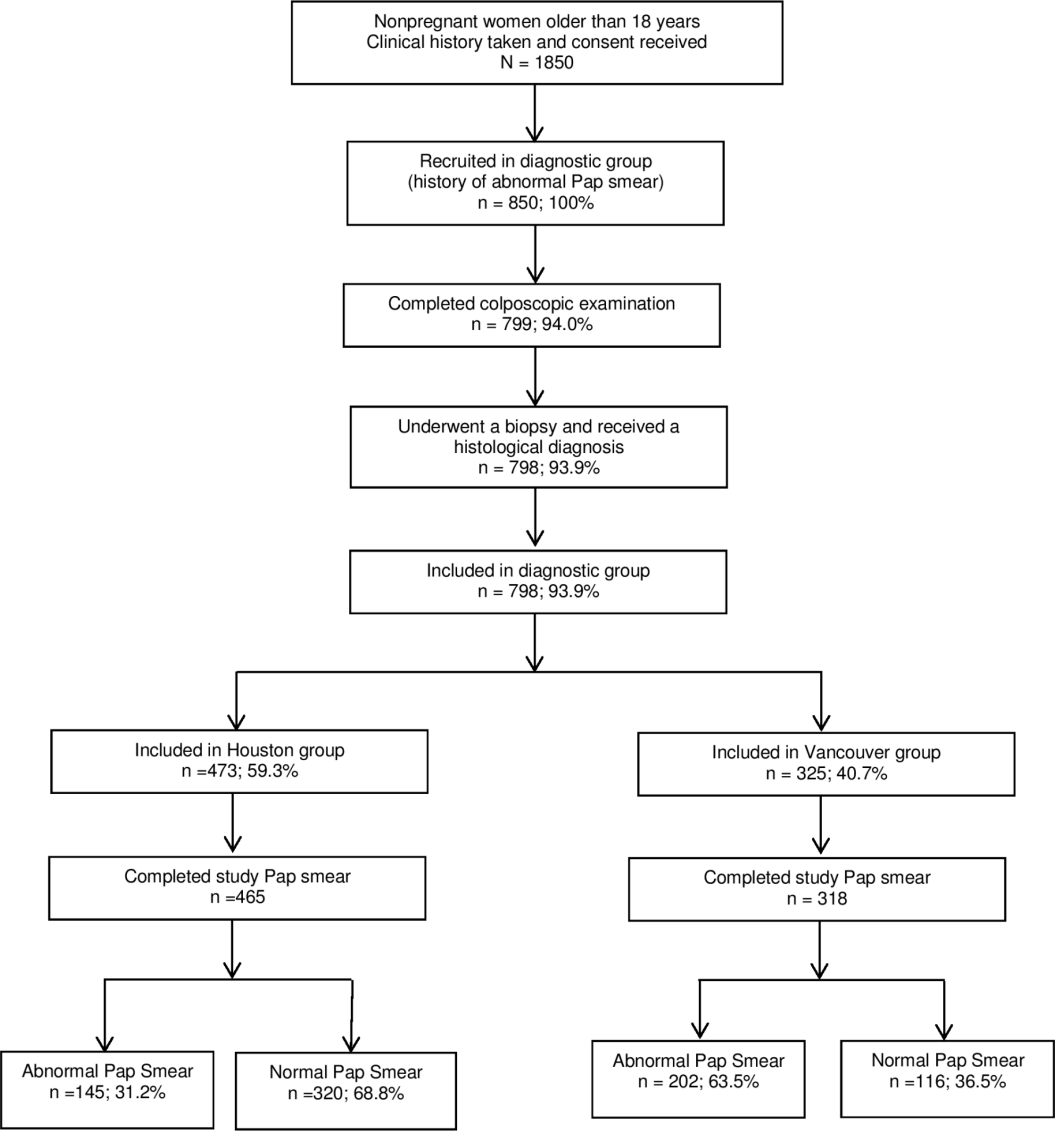
Patient inclusion criteria.

For this study, we analyzed patient data collected from an investigation of the use of optical spectroscopy, an emerging technology for the screening and diagnosis of cervical squamous intraepithelial lesions [11]. From October 1998 to November 2005, we recruited a total of 1,850 participants: 1,000 with no history of an abnormal Papanicolaou smear result (screening group) and 850 who had a recent history of an abnormal Papanicolaou smear result (diagnostic group) (**Fig 1**). Hence, this study is a retrospective analysis of patients recruited prospectively into the parent study. Given that colposcopy is typically used only in the diagnostic setting, in this report we focus only on the diagnostic group of patients.

### Study Participants

The parent study was conducted in three clinical settings: two comprehensive cancer centers in the United States and Canada and a community hospital in the United States. All participants completed an informed consent authorization, and the study was approved by the institutional review boards of all institutions affiliated with the study at the time it was conducted: The University of Texas MD Anderson Cancer Center, The University of Texas Health Science Center (UTHSC), Hermann Hospital and the Lyndon Baines Johnson Hospital Health District (both affiliated with the UTHSC), the British Columbia Cancer Agency, Rice University, and the University of British Columbia. (A protocol for the current study was submitted to the institutional review board at The University of Texas MD Anderson Cancer Center and was granted exemption.)

Participants underwent several tests associated with the current standard of care for screening and diagnosing cervical neoplasia: colposcopy, Papanicolaou smear, and human papillomavirus testing with Hybrid Capture 2. Twenty-nine colposcopists included gynecological oncologists, general obstetrician gynecologists, a family practitioner, and nurse practitioners, all of whom had at least 10 years of experience in colposcopy procedures. After applying acetic acid to the cervix as standard, the colposcopist inspected the cervix and identified the squamous columnar junction and the transformation zone. The nomenclature established by the International Federation for Cervical Pathology and Colposcopy was used to grade colposcopic lesions. Colposcopic impressions were classified as normal and benign lesions (inflammatory and metaplastic), low-grade (LG) or high-grade (HG) squamous intraepithelial lesions, or cancer.

The current standard of practice is that all sites categorized as colposcopically abnormal (LG, HG, or cancer) are biopsied. In this study, a colposcopist took one or more colposcopically directed biopsy samples of areas with abnormal colposcopic impressions according to standard practice and one or two biopsy samples of squamous and/or columnar epithelium from an area of colposcopically normal appearance. If the overall colposcopic impression was normal, biopsy samples were obtained from one or two normal sites and included both types (i.e., squamous and columnar) of cervical epithelia. Inflammatory or metaplastic changes identified by colposcopy were interpreted as benign, and biopsied tissues affected by such changes were categorized as samples of normal areas if the histopathology so indicated. All biopsy specimens were submitted to pathologists for sectioning and reading. The time interval between colposcopy and biopsy ranged from 2 to 3 minutes. There were no adverse events, and no treatments were administered between colposcopy and biopsy.

Pathologists were blinded to the colposcopic impression, test results, and medical history of the patient. All biopsy slides were read at least twice: first, on site by the local participating pathologist and then by another pathologist on the study team. If the pathologists disagreed on the pathology diagnosis, all slides were reviewed again by another study pathologist. The final histological diagnosis for each patient was based on the worst histology result among that patient’s biopsy samples. Histological diagnosis was categorized according to the World Health Organization criteria and then reclassified for analysis according to the Bethesda System for cervical cytology as follows: normal, including inflammatory lesions and atypical squamous cells of undetermined significance; low-grade squamous intraepithelial lesions (LSIL), including human papillomavirus and cervical intraepithelial neoplasia 1; high-grade squamous intraepithelial lesions (HSIL), including cervical intraepithelial neoplasia 2 and 3 and carcinoma in situ; or invasive squamous cancer. Additional data related to the research questions posed in the parent study [11] were obtained. Full details of the parent study design and the overview of study procedures can be found in reference [5].

### Statistical Analysis

We used the statistical software R, version 2.15.1, to examine frequencies and evaluate differences between patient subgroups. The demographic and clinical characteristics of patients are summarized in Table 1. Our primary analysis consisted of comparing the worst histological diagnosis among colposcopically positive sites with the worst histological finding among all sites (two to three) examined by colposcopy for each patient. This comparison provided estimates of the frequency of true positive diagnoses, as defined below, the frequency of patients with disease that would have been underdiagnosed by colposcopy, and the extent to which such disease would have been underdiagnosed.

**Table 1:** Demographic and Clinical Characteristics of Diagnostic Group Participants.

Characteristic	Diagnostic Group (%)		Houston (%)		Vancouver (%)		p-value
Total number of participants	N = 798		n = 473		n = 325		
Mean age (y)	36.4		38.2		33.8		<.001
Race							<.001
Non-Hispanic White	507	(63.5)	271	(57.3)	236	(72.6)	
African American	87	(10.9)	84	(17.8)	3	(0.9)	
Hispanic	103	(12.9)	96	(20.3)	7	(2.2)	
Native American	8	(1.0)	1	(0.2)	7	(2.2)	
Asian	63	(7.9)	15	(3.2)	48	(14.8)	
Other	30	(3.8)	6	(1.3)	24	(7.4)	
Education							<.001
Less than high school	80	(10.0)	59	(12.5)	20	(6.2)	
High school or GED	137	(17.2)	94	(19.9)	43	(13.2)	
Some college	300	(37.6)	169	(35.7)	131	(40.3)	
Bachelor	180	(22.6)	77	(16.3)	103	(31.7)	
Some graduate education	30	(3.8)	21	(4.4)	9	(2.8)	
Graduate degree	70	(8.8)	53	(11.2)	17	(5.2)	
Unknown	1	(0.1)	0	(0.0)	1	(0.3)	
Marital status							<.001
Never married	233	(29.3)	90	(19.1)	143	(44.1)	
Married	309	(38.8)	225	(47.7)	84	(25.9)	
Living in a married-like situation	76	(9.5)	32	(6.8)	44	(13.6)	
Divorced or separated	164	(20.6)	115	(24.4)	49	(15.1)	
Widowed	14	(1.8)	10	(2.1)	4	(1.2)	
Ever smoked							<.003
Yes	344	(43.2)	182	(38.6)	162	(49.8)	
No	453	(56.8)	290	(61.4)	163	(50.2)	
Number of pregnancies							<.001
None	242	(30.3)	94	(19.9)	148	45.5	
1–3	409	(51.3)	269	(56.9)	140	43.1	
More than 3	147	(18.4)	110	(23.3)	37	11.4	
Nulliparous							<.001
Yes	341	(42.7)	130	(27.5)	211	(64.9)	
No	457	(57.3)	343	(72.5)	114	(35.1)	
Menopause							<.001
No	667	(83.6)	373	(78.9)	294	(90.4)	
Yes, I am now going through it	24	(3.0)	15	(3.2)	9	(2.8)	
Yes, I have gone through it	107	(13.4)	85	(17.9)	22	(6.8)	
HPV diagnosis (by hybrid capture)							<.001
Positive							
Low risk	24	(3.0)	20	(4.3)	4	(1.2)	
High risk	323	(40.8)	118	(25.2)	205	(63.5)	
Both	60	(7.6)	19	(4.1)	41	(12.7)	
Negative	384	(48.5)	311	(66.5)	73	(22.6)	

GED, general equivalency degree; HPV, human papillomavirus

Data are n (%) unless otherwise specified.

We used two thresholds for positive diagnosis based on histology: LSIL and HSIL. For positive (abnormal) colposcopy diagnosis, we use the LG colposcopy threshold. The worst histology result among all the biopsy samples of each patient was considered the true histological diagnosis. We also analyzed the diagnostic patient subgroup according to geographic location (Houston, TX, USA and Vancouver, BC, Canada) because of the possible effects from precancer prevalence and threshold for positive diagnosis. We analyzed the effect of the study Papanicolaou smear result on the diagnostic accuracy of colposcopy. Patients were categorized as having a positive study Papanicolaou smear if they had abnormal results such as atypical squamous cells of undetermined significance or a worse condition (e.g., LSIL, HSIL, or cancer). We used the Pearson chi-square test for independence to assess the significance of differences in detection of precancer between the subgroups. Because of the low numbers of patients with HSIL or cancer in some subgroups, those patients were grouped together with patients with diagnoses of LSIL for comparison with patients whose condition was diagnosed as normal.

The evaluation of the accuracy of colposcopy was based on the worst histological diagnosis among each patient’s colposcopically positive sites. Thus, a patient with HSIL only at colposcopically negative sites would have a colposcopic diagnosis of normal despite actually having HSIL precancer, even if they had colposcopically positive sites where the histological diagnosis was lower than HSIL. In clinical practice, any site classified by colposcopy as LG is considered positive, so all of our analyses were based on this threshold for colposcopy. Patient results were classified as follows:

- Colposcopic and histological findings that were all negative were categorized as true negative;
- Patients with at least one colposcopically positive site and only negative histological findings were categorized as false positive.
- Patients who had a positive histological diagnosis for at least one colposcopically positive site were categorized as true positive.
- Patients with results categorized as false negative comprised two groups; in one group, all sites were colposcopically negative and at least one of those sites was histologically positive; the second group had a histologically negative result(s) at a colposcopically positive site(s) and a histologically positive result at a colposcopically negative site(s).

Thus, false negatives may have had colposcopically positive sites but histology was negative at those sites and positive at colposcopically negative sites. The proportion of patients in this group is less than or equal to the true proportion of actual false negatives in our patient sample, since there may have been patients who truly had disease and it was not detected by any of our biopsies. Such patients would have been incorrectly classified as true negatives (if the colposcopy was all negative) or false positives (if there was positive colposcopy). We determined the sensitivity of colposcopy by calculating the ratio of true positives to the total number of patients with positive histology findings: true positives/(true positives + false negatives). We determined specificity by calculating the ratio of true negatives to the total number of patients with negative histology findings: true negatives/(true negatives + false positives). Exact 95% confidence intervals (CIs) were calculated.

## Results

The results of the study are based on 798 of the original 850 patients in the diagnostic group (**Fig 1**). As in our previous analysis, patients without at least one recorded colposcopy result (51 patients) were excluded from our analysis. In addition, one patient whose biopsy samples were unreadable was also excluded from our analysis. A less strict inclusion criteria resulted in one more patient than Cantor et al [5].

With positive-diagnosis thresholds of LSIL for histology, the sensitivity of colposcopy decreased from 87.0%, as determined in our previous assessment, to 74.0%, while specificity remained the same. The positive and negative likelihood ratios were 1.7 and 0.5, respectively, compared to our previous estimates of 2.024 and 0.213 [5]. In the Houston clinics, sensitivity was 61.7% and specificity was 66.7%, compared to sensitivity of 85% and specificity of 29% in the Vancouver clinic. The likelihood ratios for the two cities were similar: the positive and negative likelihood ratios were 1.8 and 0.6, respectively, in Houston and 1.2 and 0.5, respectively, in Vancouver. Detailed results and confidence intervals are given in **Table 2.** An example that illustrates the calculation of the aforementioned quantities is provided in the Appendix **(S1 Appendix).**

**Table 2:** Summary of Results: Colposcopy sensitivities, specificities, positive and negative likelihood ratio values.

Histological diagnosis	Sensitivity (%)	95% CI	Specificity (%)	95%CI	LR+	LR–
Diagnostic group						
(n = 798)						
HSIL and worse	87.0	81.8–90.9	45.0	40.8–49.2	1.6	0.3
LSIL and worse	74.0	67.4–76.1	56.4	51.2–61.5	1.7	0.5
Houston diagnostic group						
(n = 473)						
HSIL and worse	85.6	75.1–92.2	55.9	50.9–60.8	1.9	0.3
LSIL and worse	61.7	54.6–68.3	66.7	60.6–72.2	1.8	0.6
Vancouver diagnostic group						
(n = 327)						
HSIL and worse	87.7	81.3–92.3	19.4	13.9–26.3	1.1	0.6
LSIL and worse	85.3	79.9–89.6	29.0	20.6–39.1	1.2	0.5
Houston positive study Papanicolaou smear^†^						
(n = 145)						
HSIL and worse	87.5	76.3–94.1	33.3	23.5–44.8	1.3	0.4
LSIL and worse	79.8	70.8–86.7	55.6	38.3–71.7	3.1	0.6
Houston negative study Papanicolaou smear^†^						
(n = 320)						
HSIL and worse	72.7	39.3–92.7	61.8	56.1–67.2	1.9	0.4
LSIL and worse	39.1	29.3–49.9	68.0	61.4–73.9	1.2	0.9
Vancouver positive study Papanicolaou smear^†^						
(n = 202)						
HSIL and worse	86.0	77.9–91.5	21.6	13.8–31.9	1.1	0.7
LSIL and worse	85.8	78.9–90.8	33.3	21.5–47.6	1.3	0.4
Vancouver negative study Papanicolaou smear^†^						
(n = 116)						
HSIL and worse	92.3	78.0–98.0	16.9	9.6–27.5	1.1	0.5
LSIL and worse	83.8	73.0–91.0	23.8	12.6–39.8	1.1	0.7

LG threshold for positive diagnosis by colposcopy was used; LR+ = positive likelihood ratio, LR– = negative likelihood ratio;

^†^Obtained as part of the study.

**Tables 3–9** show comparisons of the worst histological diagnoses among colposcopically positive sites with worst overall histological diagnoses for various subgroups of patients. These comparisons allowed us to determine the percentage of patients with disease that would have been underdiagnosed by colposcopy and the numbers of patients with colposcopically positive, histologically negative sites and colposcopically negative, histologically positive sites. The results for these patients would be false negative if biopsies were taken only at colposcopically positive sites. The size of this group was underestimated in our previous analysis [5].

**Table 3:** Comparison of worst histological diagnosis among sites diagnosed as positive by colposcopy with worst overall histological diagnosis for the 798 diagnostic group patients.

Worst histological diagnosis among positive colpo sites	Worst histological diagnosis among all colpo sites (%, 95% CI):								Total no. of patients
	Normal		LSIL*		HSIL^†^		Cancer		
No positive colpo sites	207	(56, 51–62)	48	(24, 18–31)	4	(2, .6–5)	0		259
Normal	160	(44, 38–49)	43	(21, 16–28)	17	(7, 5–12)	0		220
LSIL			109	(55, 47–61)	9	(4, 2–8)	0		118
HSIL					196	(87, 81–91)	0		196
Cancer							5	100	5
Total no. of patients	367		200		226		5		798

LSIL, Low-grade squamous intraepithelial lesions; HSIL, high-grade squamous intraepithelial lesions; CI, confidence interval

No colpo positive sites, all patient sites diagnosed as normal by colposcopy.

**Table 4:** Comparison of worst histological diagnosis among sites diagnosed as positive by colposcopy with worst overall histological diagnosis for the 473 Houston diagnostic patients.

Worst histological diagnosis among positive colpo sites	Worst histological diagnosis among all colpo sites (%, 95% CI):								Total no. of patients
	Normal		LSIL		HSIL		Cancer		
No colpo positive sites	178	(67, 61–72)	44	(34, 26–43)	3	(4, 1–13)	0		225
Normal	89	(33, 28–39)	28	(21, 15–30)	4	(6, 2–15)	0		121
LSIL			58	(45, 36–54)	4	(6, 2–15)	0		62
HSIL					60	(84, 74–92)	0		60
Cancer							5	(100)	5
Total no. of patients	267		130		71		5		473

LSIL, Low-grade squamous intraepithelial lesions; HSIL, high-grade squamous intraepithelial lesions; CI, confidence interval

No colpo positive sites, all patient sites diagnosed as normal by colposcopy.

**Table 5:** Comparison of worst histological diagnosis among sites diagnosed as positive by colposcopy with worst overall histological diagnosis for the 325 Vancouver diagnostic patients.

Worst histological diagnosis among positive colpo sites	Worst histological diagnosis among all colpo sites (%, 95% CI):							Total no. of patients
	Normal		LSIL		HSIL		Cancer	
No colpo positive sites	29	(29, 21–39)	4	(6, 2–15)	1	(1, 0–4)	0	34
Normal	71	(71, 61–79)	15	(21, 13–33)	13	(8, 5–14)	0	98
LSIL			51	(73, 61–82)	5	(3, 1–8)	0	56
HSIL					136	(88, 81–92)	0	136
Cancer							0	0
Total no. of patients	99		70		155		0	325

LSIL, Low-grade squamous intraepithelial lesions; HSIL, high-grade squamous intraepithelial lesions; CI, confidence interval

No colpo positive sites, all patient sites diagnosed as normal by colposcopy.

**Table 6:** Comparison of worst histological diagnosis among sites diagnosed as positive by colposcopy with worst overall histological diagnosis for the 145 Houston diagnostic patients with a positive Papanicolaou smear diagnosis.

Worst histological diagnosis among positive colpo sites	Worst histological diagnosis among all colpo sites (%, 95% CI):								Total no. of patients
	Normal		LSIL		HSIL		Cancer		
No colpo positive sites	20	(56, 38–72)	7	(16, 7–30)	2	(3, 0.6–13)	0		29
Normal	16	(44, 28–62)	10	(22, 12–37)	3	(5, 1–15)	0		29
LSIL			28	(62, 47–76)	3	(5, 1–15)	0		31
HSIL					51	(87, 74–94)	0		51
Cancer							5	(100)	5
Total no. of patients	36		45		59		5		145

LSIL, Low-grade squamous intraepithelial lesions; HSIL, high-grade squamous intraepithelial lesions; CI, confidence interval

No colpo positive sites, all patient sites diagnosed as normal by colposcopy.

**Table 7:** Comparison of worst histological diagnosis among sites diagnosed as positive by colposcopy with worst overall histological diagnosis for the 320 Houston diagnostic patients with a negative study Papanicolaou smear diagnosis.

Worst histological diagnosis among positive colpo sites	Worst histological diagnosis among all colpo sites (%, 95% CI):							Total no. of patients
	Normal		LSIL		HSIL		Cancer	
No colpo positive sites	155	(68, 61–74)	36	(44, 34–56)	1	(9, 0.5–43)	0	192
Normal	73	(32, 26–39)	18	(22, 14–33)	1	(9, 0.5–43)	0	92
LSIL			27	(33, 23–45)	1	(9, 0.5–43)	0	28
HSIL					8	(73, 39–93)	0	8
Cancer							0	0
Total no. of patients	228		81		11		0	320

LSIL, Low-grade squamous intraepithelial lesions; HSIL, high-grade squamous intraepithelial lesions; CI, confidence interval

No colpo positive sites, all patient sites diagnosed as normal by colposcopy.

**Table 8:** Comparison of worst histological diagnosis among sites diagnosed as positive by colposcopy with worst overall histological diagnosis for the 202 Vancouver diagnostic patients with a positive study Papanicolaou smear diagnosis.

Worst histological diagnosis among positive colpo sites	Worst histological diagnosis among all colpo sites (%, 95% CI):							Total no. of patients
	Normal		LSIL		HSIL		Cancer	
No colpo positive sites	18	(33, 21–48)	1	(3, 0.2–17)	1	(0.9, 0.05–6)	0	20
Normal	36	(67, 52–79)	9	(26, 14–45)	10	(9, 5–16)	0	55
LSIL			24	(71, 52–84)	5	(4, 2–10)	0	29
HSIL					98	(86, 78–92)	0	98
Cancer							0	0
Total no. of patients	54		34		114		0	202

LSIL, Low-grade squamous intraepithelial lesions; HSIL, high-grade squamous intraepithelial lesions; CI, confidence interval

No colpo positive sites, all patient sites diagnosed as normal by colposcopy.

**Table 9:** Comparison of worst histological diagnosis among sites diagnosed as positive by colposcopy with worst overall histological diagnosis for the 116 Vancouver diagnostic patients with a negative study Papanicolaou smear diagnosis.

Worst histological diagnosis among positive colpo sites	Worst histological diagnosis among all colpo sites (%, 95% CI):							Total no. of patients
	Normal		LSIL		HSIL		Cancer	
No colpo positive sites	10	(24, 13–40)	3	(9, 2–24)	0		0	13
Normal	32	(76, 60–87)	6	(17, 7–34)	3	(8, 2–22)	0	41
LSIL			26	(74, 56–87)	0		0	26
HSIL					36	(92, 78–98)	0	36
Cancer							0	0
Total no. of patients	42		35		39		0	116

LSIL, Low-grade squamous intraepithelial lesions; HSIL, high-grade squamous intraepithelial lesions; CI, confidence interval

No colpo positive sites, all patient sites diagnosed as normal by colposcopy.

In **Tables 3–9**, the entries along the diagonal correspond to cases of correct diagnosis by colposcopy, i.e., the worst histology among colposcopically positive sites is the same as the worst histology overall; thus, the entries above the diagonal correspond to cases of underdiagnosis.

**Table 3** shows a comparison of the worst histological diagnoses among colposcopically positive sites with the worst histological diagnoses among all sites examined by colposcopy for all 798 patients. Of the 200 patients with a worst histological diagnosis of LSIL, 46% (95% CI 39–53%) would have had their condition underdiagnosed as normal by colposcopy. Of the 226 patients with a histological diagnosis of HSIL, 9% (95% CI 6–14%) would have had their condition misdiagnosed as normal by colposcopy. An additional 4% (95% CI 2–8%) of patients with a histological diagnosis of HSIL would have had their condition underdiagnosed as LSIL by colposcopy.

We also analyzed diagnostic patient subgroups by location, as shown in **Tables 4** and **5**. Of the 130 Houston patients with a histological diagnosis of LSIL, 55% (95% CI 46–64%) would have had their condition underdiagnosed as normal, compared to 27% (95% CI 18–40%) of the 70 Vancouver patients with a histological diagnosis of LSIL. Similarly, among patients with a histological diagnosis of HSIL, 10% (95% CI 4–20%) of 71 Houston patients, compared to 9% (95% CI 5–15%) of 155 Vancouver patients, would have their condition underdiagnosed as normal. An additional 6% (95% CI 2–15%) of the 71 Houston patients compared to 3% (95% CI 1–8%) of the 155 HSIL Vancouver patients would have had their condition underdiagnosed as LSIL. These differences between the Houston and Vancouver diagnostic patient subgroups were statistically significant (P < 0.001).

**Tables 6–9** show the results for patients with positive and negative study Papanicolaou smear results in Houston and Vancouver. In Houston, among the 45 patients with a positive study Papanicolaou smear result and histological diagnosis of LSIL, 38% (95% CI 24–54%) would have had their condition underdiagnosed as normal by colposcopy compared to 29% (95% CI 15–48%) of the 34 patients in Vancouver. Among the 59 patients with a positive study Papanicolaou smear result and histological diagnosis of HSIL in Houston, 8% (95% CI 3–19%) would have had their condition underdiagnosed as normal compared to 10% (95% CI 5–17%) of the 114 patients in Vancouver. An additional 5% (95% CI 1–15%) of the 59 patients in Houston would have had their condition underdiagnosed as LSIL compared to 4% (95% CI 2–10%) of the 114 patients in Vancouver. These differences were statistically significant (P<.02).

Among the 81 patients in Houston with a negative study Papanicolaou smear result and histological diagnosis of LSIL, 67% (95% CI 55–77%) would have had their condition underdiagnosed as normal by colposcopy compared to 25% (95% CI 13–44%) of the 35 patients in Vancouver. Of the 11 patients with a histological diagnosis of HSIL in Houston, 18% (95% CI 3–52%) would have had their condition underdiagnosed as normal compared to the 8% (95% CI 2–22%) of the 39 patients in Vancouver. An additional 9% (95% CI 0.4–43%) of the Houston patients would have had their condition underdiagnosed as LSIL. These differences were statistically significant (P<.001).

## Discussion

Our reanalysis of the accuracy of diagnostic colposcopy showed that colposcopy is less sensitive than estimated in previous studies. However, our results are within the ranges of other estimates in the literature [2–10, 13, 14]. In agreement with recent studies showing that colposcopy performs well as an adjunct to biopsy, our findings support the use of colposcopy as a diagnostic aid following an abnormal Papanicolaou smear result and in guiding biopsy [7, 15, 16].

Our results are also consistent with the earlier analysis by Cantor et al. [5]. However, the sensitivity of colposcopy in the current study was lower, because we took into account the histologically positive (false negative) sites that would have been missed by colposcopy. The present analysis reported here was more precise, because we analyzed biopsy samples from both colposcopically normal and colposcopically abnormal sites for each patient. This approach can be thought of as providing an upper bound on sensitivity, since some patients with disease may have been missed and wrongly included in the true negative group.

Our study, however, is not without limitations. This is one study conducted in two locations which can limit generalizability. However, our results are within the range of other estimates. In addition, we conducted the study at various hospitals which provided ethnic diversity by capturing African-American and Hispanic patients in Houston and Native American and Asian patients in Vancouver. Our analysis also relied on multiple biopsies of the cervix, including normal and abnormal areas. The analysis was therefore limited by the accuracy of colposcopy in selecting sites of biopsy.

Colposcopy had greater sensitivity and lower specificity in the Vancouver clinic than in the Houston clinics. Positive predictive values were higher in Vancouver than in Houston, while negative predictive values were lower than in Houston. However, the positive and negative likelihood ratios for the two cities were similar. Patients with lesions are not referred for colposcopy in Vancouver unless the Papanicolaou smear reading indicates HSIL, whereas the criterion for referral in Houston is presence of atypical squamous cells of undetermined significance. Therefore, some of the differences in the accuracy of colposcopy may be attributable to differences in the prevalence of HSIL between the two locations (much higher in Vancouver than in Houston) in the diagnostic setting. In Vancouver, patients diagnosed with LSIL on the basis of a Papanicolaou smear are treated as the normal patient group and placed on a “watchful waiting” regimen, and only patients diagnosed with HSIL on the basis of a Papanicolaou smear are evaluated. Some studies have shown that this practice may prevent overtreatment [13] while others suggest that sending LSIL patients for immediate colposcopy may avoid the anxiety associated with repeated Papanicolaou smears [17].

Thus, the Vancouver group consisted only of patients diagnosed with HSIL on the basis of a Papanicolaou smear, while the Houston diagnostic group included patients diagnosed with lower-grade abnormalities on the basis of a Papanicolaou smear. The higher prevalence of cervical precancer in the Vancouver patient group may explain the higher colposcopy specificities than those in Houston. Theoretically, sensitivity and specificity should not depend on the prevalence of the true underlying disease status. However, as noted by Fletcher et al., several disease characteristics, such as stage and severity, may be related both to the sensitivity and specificity of a test and to prevalence, because different kinds of patients are found in high- and low-prevalence situations [18]. This aspect of clinical practice may affect the diagnostic accuracy of colposcopy, since colposcopists may look more carefully for lesions in a population with higher disease prevalence. A study by Luesley and Downey [19], which supports a “watchful waiting regimen,” also underscores the importance of significant colposcopic training. Another factor may be that in Houston, the colposcopies were conducted primarily by nurse practitioners, whereas in Vancouver they were usually conducted by gynecologic oncologists, who have had more advanced training in recognizing abnormal areas in the cervix. However, recent studies have shown that when significant training is provided, colposcopic performance of nurse practitioners and physicians is comparable [8].

## Conclusions

By comparing the worst histology result among colposcopically positive sites to the worst histology result among all sites biopsied for each patient, we were able to more validly assess the number of patients with cervical intraepithelial neoplasia that would have been missed by colposcopy, whereas other methods may overestimate the diagnostic accuracy of colposcopy. Appropriate measures of diagnostic test accuracy should reflect actual rather than hypothetical usage in the clinical setting.

The potential impact of underdiagnosis by colposcopy has been documented. Given that we cannot sample the entire cervix, this does emphasize the significance of multiple biopsies and the clinical importance of colposcopy in selecting sites for biopsy. We recommend the use of confirmatory biopsies. Colposcopy in conjunction with human papillomavirus testing may be a potential alternative. A recent study has shown that the diagnostic accuracy of colposcopy is also improved when colposcopy is used in conjunction with spectroscopy [20].

Although not all colposcopy diagnoses in our study were accurate, overall, colposcopy performed relatively well. However, the limitations of colposcopy leave room for development of technologies that would provide a real-time diagnosis [21]. They also serve as a lesson on the importance of using a valid methodology for assessing diagnostic accuracy.

## Supporting Information

S1 Appendix Calculating Summary Diagnostic Information: Sensitivities, Specificities, Positive and Negative Predictive Values, and Likelihood ratios using Table 3 information. (DOCX)

S1 Table STARD checklist for reporting of studies of diagnostic accuracy. (DOCX)
