# Supplementary material for: An Alternative Approach for Estimating the Accuracy of Colposcopy in Detecting Cervical Precancer
Source: PLoS One. 2015 May 11;10(5):e0126573. doi: 10.1371/journal.pone.0126573 (PMC4427274; doi:10.1371/journal.pone.0126573)
Supplement: S1 Appendix — (DOCX) [file pone.0126573.s001.docx]

**S1 Appendix.** Calculating Summary Diagnostic Information: Sensitivities, Specificities, Positive and Negative Predictive Values, and Likelihood ratios using Table 3 information

Using LSIL threshold for positive histology:

|  |  | Histology | |  |  |
| --- | --- | --- | --- | --- | --- |
|  |  | Histo + | Histo - | |  |
| Colposcopy | Colpo + | TP =109+9+196+5 = 319 | FP = 160 | | PPV = TP/ Colpo +  = 319/ 319 + 160  = 0.67 |
|  | Colpo − | FN = 48+4+43+1 = 112 | TN = 207 | | NPV = TN/Colpo –  = 207/112+207  = 0.65 |
|  | LR+ = TPR/ FPR  = 1.70 | Sens = TPR= TP/ TP+FN  = 74.1% | 1-Spec = FPR  = 43.6% | | Total = 798 |
|  | LR − = FNR/TNR  = 0.46 | 1-Sens = FNR  = 25.9% | Spec = TNR = TN/ TN + FP  = 56.4% | | Accuracy = TP + TN /  Total  = 319 +207/798  = 65.9% |

TP, True positive; FN, False negative; FP, False positive; TN = True negative;

Sens, Sensitivity; Spec, Specificity, PPV, positive predictive value;

NPV, negative predictive value; LR+, positive likelihood ratio; LR-, negative likelihood ratio

LSIL, Low-grade squamous intraepithelial lesions

Using HSIL threshold for positive histology:

|  |  | Histology | |  |  |
| --- | --- | --- | --- | --- | --- |
|  |  | Histo + | Histo - | |  |
| Colposcopy | Colpo + | TP =196+5 = 201 | FP = 160+43+109 = 312 | | PPV = TP/ Colpo +  = 201/ 201+312  = 0.39 |
|  | Colpo − | FN = 4+17+9 = 30 | TN = 207+48 =255 | | NPV = TN/Colpo –  = 255/30+255  = 0.89 |
|  | LR+ = TPR/ FPR  = 1.58 | Sens = TPR= TP/ TP+FN  = 87.0% | 1-Spec = FPR  = 55.0% | | Total = 798 |
|  | LR − = FNR/TNR  = 0.29 | 1-Sens = FNR  = 13.0% | Spec = TNR  = TN/ TN + FP  = 45.0% | | Accuracy = TP + TN /  Total  = 201+255/798  = 65.9% |

TP, True positive; FN, False negative; FP, False positive; TN = True negative;

Sens, Sensitivity; Spec, Specificity, PPV, positive predictive value;

NPV, negative predictive value; LR+, positive likelihood ratio; LR-, negative likelihood ratio

HSIL, high-grade squamous intraepithelial lesions
